# Facio Maxillary Surgery and Oral Focal Infections

**Published:** 1919

**Authors:** 


### FACIO-MAXILLARY SURGERY AND ORAL FOCAL INFECTIONS

(Editor's Note : Questionnaire prepared by Captain A. W. Nodine, American Red Cross, and Major Kazarjam of the British Armies in France. Replies compiled by Captain Nodine.)

### I. Wounds of the Maxillae with Loss of Substance

Q. 1. — **What methods may be used to immobilize disarticulated maxillae ?**

A. — *a*) With teeth present in the fragments of the disarticulated maxillae, and bodily fracture with slight loss of substance : a cast or swagged metal splint cemented on to the reduced fragments, held immobile by adjustable headgear, by means of which traction, pressure backward, or laterally, may be accomplished. Cusps of teeth in lower jaw, and pressure of lower jaw may be utilized to the same end. Possibly the flange attached to lower splint may help to retain the fragments in proper relation to the midline of the body and in proper condition.

*b*) In those cases in which there are no teeth in the fragments : vulcanite plates may be attached to the adjustable headgear, by means of which pressure can be applied in the [direction desired. These vulcanite plates, preferably removable, will mould the tissues and retain the periosteum in normal contour.

*c*) In cases in which some teeth are present, and some of the fragments without teeth : to immobilize such a condition, a cast or swagged metal or vulcanite splint cemented on to the solid fragment, to which is attached a vulcanite or metal pad or saddle holding the loose fragments immobile and in proper place.

*d*) In fractures involving the antrum, if the antrum is opened and a large part of the wall gone : construct a vulcanite body to maintain the contour of the face and prevent the attachment of a scar tissue to the edges of the antrum wall, holding it to the splints cemented to the solid teeth. Secure adequate drainage and frequent irrigation. If the antrum is badly affected, secure adequate drainage, preferably into the inferior meatus of the nose.

*e*) In cases in which the fracture involves the malar bone or the orbit : the malar bone may be wired in reduction, even if the wire must be removed later. The fragments may be retained at least until fibrous union occurs. The same may be done with the edge of the orbit and zygoma. Fragments of the malar bones and zygoma may be reduced and held by vulcanite, metal or compound pad or saddle within the mouth, producing pressure upward and forward, providing that there is no lesion of the mucous membrane, and held immobile by adjustable headgear or attached to upper splint.

*f*) In those cases in which there is extensive loss of tissue between the maxillae and the cranium : surgical interference can hope for very little in regard to stabilizing. The best that surgical intervention has so far accomplished in these cases is to so prepare the tissues that suitable prosthetic appliances may be used.

Q. 2. — **What percentage of disarticulated maxillae attain bony union ?**

A. — *a*) The greatest success has been claimed for those cases of disarticulated maxillae in which there is no large amount of lost bony tissue, and in which immobilization has been accomplished at an early stage.

*b*) In those cases in which there has been considerable loss of bony tissue, but not a distinct cleft between the maxillary bones and the cranium, the success has not been so great.

*c*) In those cases in which there has been a distinct bony cleft between the superior maxillary bones and the cranium, results have not been all that might be desired.

Remark. — A mobile or un-united upper jaw is a rare occurrence, if treatment is begun early, regardless of the extent of the injury.

The earlier the treatment is begun in these cases, the more certain and complete will be the success.

Q. 3. — **In the restoration of lost substances, what transplants and grafts give the best results ?**

A. — *a*) On account of the septic conditions which accompany openings into the nasal, frontal and maxillary sinuses, bone or cartilage grafts are made under difficulties not found elsewhere. Disregarding the repair of defects in the nose, lost soft tissue is best repaired by means of pedicle grafts taken from the temporal or clavicular regions, neck, arm or abdomen, followed by skin grafts when desired, or when skin has not been included in the pedicle graft.

*b*) Decalcified bone may also be used to fill gaps or depressions in which it is not desirable or advisable to use the pedicle graft.

Q. 4. — **In what cases are direct transplants and grafts indicated ?**

A. — Direct grafts or transplants are indicated in those situations

*a*) in which sepsis has entirely disappeared,

*b*) in which a liberal blood supply is present, and

*c*) in which there is a reasonable amount of surety for the establishment of a good circulation in graft.

Indirect grafts or transplants are indicated in those locations in which it is difficult to obtain or to establish good circulation by means of a direct graft.

The indirect grafts should not be allowed to remain in the temporary site more than a month or two before being directly placed in the gap desired to be bridged.

Q. 5. — **What advantages have prosthetic appliances in the restoration of lost substance?**

A. — Prosthetic appliances have the following advantages in the restoration of lost substances :

*a*) Lost substance may be restored in a very much shorter time than that required for surgical intervention.

*b*) Prosthetic appliances may be employed where it is impossible and difficult to accomplish the desired result by surgical intervention.

*c*) Prosthetic appliances may restore a certain amount of function impossible to obtain by surgical means.

*d*) Prosthetic appliances may supplement and make possible certain surgical operations which would not otherwise be successful without them.

Q. 6. — **What materials are congenial to the tissue in the restoration by prosthetic appliances ?**

A. — Platinum, gold, silver, tin, cast aluminium, highly polished vulcanite, gutta percha.

### II. Wounds of Mandible with Loss of Substance

Q. 1. — **In the restoration of one-half or more of the mandible, including the condyle, what may surgical intervention expect to accomplish ?**

A. — Surgical restoration of one-half or more of the mandible, including the condyle, has so far fallen short of anticipation. The results hitherto obtained have not been very successful. It has been proposed, and it has been tried, to restore this half of the mandible, including the condyle, by the use of a replica in boiled bone, ivory and silver, but these attempts and these suggestions do not promise the functional restoration as desired. But surgical intervention may so prepare the tissues for the reception of prosthetic appliances that they may impart and restore a certain amount of function.

If there remains, attached to the free end of the fragment, muscle which can exert a certain amount of pressure, the mandible deprived of the posterior segment may still functionate in a fairly good manner. The destruction of a part or the whole of the ramus of the mandible may not affect greatly the activity of the jaw for the purposes of mastication, provided that the normal side is free from deformity and is moderately well supplied with teeth. Surgical interference, when restoring the lost soft tissues, may utilize grafts taken from either the temporal muscle or from the muscles and tissues of the neck and clavicular regions, and sometimes from the arm and abdomen. By such plastic operations, surgical intervention may close the gap with soft tissues, restoring a part of the contour of the floor and boundaries of the buccal cavity, and thereby may greatly facilitate the application of prosthetic appliances.

Q. 2. — **The loss of the anterior third mandible may be best restored by what methods ?**

A. — The following methods have been used to restore the anterior third part of the mandible :

Immobilization of the two halves of the jaw and the construction of such prosthetic appliances as will attempt to overcome contraction and distortion of the tissues until the disappearance of sepsis. An effort is made to restore the lost soft parts by suitable grafts of tissue taken from the neck, chest, arm or abdomen. These grafts must be at least one-third larger than the space to be bridged, in order to allow for contraction. They must be made in such a way as to restore the contour of the chin, and also to form epithelial floor for the mouth. Sufficient tissue must be provided, first for the nourishment of the bone graft, and second, so that, in making a bed for the bone graft, entrance into the buccal cavity may be avoided. These grafts, while preserving the contour, are kept from contracting by suitable vulcanite or metal pads.

These bone grafts may be taken from the following bones and locations :

(1) Crest of the ilium.

(2) A rib.

(3) Osteo-periostic, taken from the internal surface of the tibia, preferably the upper third. These grafts are about two or three mm. thick. They are taken by means of an operating knife, a chisel 1 cm. wide, and a mallet under the fibro-periostic part of the bone fragments. (This method may also be used in restoring the lateral third of the mandible). The bone is not sutured, but suture is performed on the four cutaneous bands which act as a support. Suitable drains are maintained by means of subcutaneous drains, over which the dressing is applied. The success of these grafts, in common with other types, depends upon :

*a*) Total asepsis of the graft and wound.

*b*) Complete closure of the wound, and covering up of the graft with healthy tissue and teguments.

*c*) Close contact between the graft and the fragments.

*d*) Complete and lengthy immobilization of the segments and the graft. (Delagénière).

(4) Inner surface of the tibia.

(5) Part of the clavicle.

(6) Boiled bone.

(7) Pedicle grafts taken from the rib, clavicle or sternum.

Q. 3. — **With the loss of bone and soft tissues between the ramus and the symphysis, what grafts and transplants promise the best results ?**

A. — The statements made in regard to bone grafts in answer to the previous question apply also to this.

The grafts most suitable for replacing lost bone in the lateral half of the jaw are :

(1) A rib graft.

(2) Inner surface of the tibia.

(3) Crest of the ilium.

(4) The clavicle.

(5) Rib cartilage.

(6) Pedicle graft. This consists of a section of bone which may be taken with the soft tissue attached from either one or both halves of the mandible, then slid over to bridge the gap and fixed with the wire of screws. (Cole).

A second type of pedicle graft may be made from the clavicle, sternum or rib, and fixed in the same manner. Pedicle grafts have two marked advantages :

*a*) A well-nourished flap with a good pedicle can be implanted in a granulating surface with better prospects of healing quickly and with more surety than will be afforded by a free graft.

*b*) Such a flap will serve to fill up a gap left in the soft tissues. (Groves).

(7) Boiled bone.

(8) Ivory.

(9) After the preliminary preparation of the mandible, similar in all bone grafting operations, a motor saw is used to cut a slot in a lower inferior border of the segments extending one-half inch to one inch and a half back from the end of each fragment, and about one-half inch deep. An osteoma is then driven into the saw-cut, and a greenstick fracture produced, making a wedge-shaped gap for the reception of the graft. The graft is then made by resecting sufficient of a rib to fill the gap and extend into the end of the saw-cut groove. The rib is then split on the flat, in order that the endosteal surface may be bathed in lymph. Half of the graft is then driven into the slots in the fragments, the smooth side of the rib facing towards the mouth cavity,thus leaving a sunken surface somewhat below the outer surface of the jaw. This area is then filled in by laying a piece of the other half of the rib in the gap with the smooth side out. The fragments and grafts are now fastened solidly in place with kangaroo tendon or silver wire passed through drilled holes. (Gallie and Robertson).

### III. — Oral Focal Infections

Q. 1. — **What effect has pyorrhea alveolaris on the organs and tissues of the body and on the blood and the nervous system ?**

A.—Pyorrhea alveolaris and infective gingiva have the following effects :

(1) The outpouring of lymph and leucocytes to combat the destructive processes of the infection is a constant drain upon the system's resistive powers. It lowers the resisting powers of the individual so infected in two ways : —

*a*) The individual so infected has his resistance lowered by nature's effort to repair and to combat the destructive processes in the diseased tissues.

*b*) The absorption of pathogenic micro-organisms and toxins from these pathological tissues.

(2) Examination of the blood of patients who have marked pyorrhea and gingival infections shows a condition resembling that found in puerperal fever and advanced carcinoma.

(3) It has not as yet been recognized that micro-organisms from gingival infections produce any specific organic or systemic disease or disorder.

(4) But gingival infections, among other infections from other organs and other locations, are recognized as being one of the possible and common sources of infection that produce directly or indirectly, or contribute to the cause of the following pathological conditions :

*a*) Serious, grievous and dangerous diseases of the heart : as endocarditis, myocarditis, pericarditis, pancarditis.

*b*) Serious and insidious diseases of the nervous system :

1. Indirectly by producing nutritional disturbances, anemia, and toxic states in which the nervous system suffers, as nerve exhaustion, hysteria, hypochondria, neurasthenia, psychasthenia,

2. Directly, gingival infection has been held by some to have caused cases of (1) epilepsy, (2) bulbar paralysis, (3) lesions in the brain and spinal cord (the infection following the lymph spaces of the nerve trunks), (4) neuritis.

(5) *a*) Pernicious anemia. *b*) Anemias of the secondary type, including streptococcal, pneumococcal, and other infections of the blood. *c*) Leukemia.

(6) Arthritis.

(7) Chronic infections are almost invariably associated with glandular syndromes. These infections are usually focal in character and seem to occur with the same relative frequency in insufficiency of the thyroid, pituitary glands and adrenals.

*a*) Thyroid intoxication.

*b*) Thyroid enlargement.

*c*) Thyroid deficiency.

(8) Nephritis.

(9) Skin diseases.

*a*) Those related to : joint affections ; erythema nodosum ; erythema multiforme ; gouty eczema ; psoriasis.

*b*) Those neuropathic in character: lichen planus; lichen simplex (Vedal) ; herpes simplex ; neurotic eczema ; herpes zoster ; alopecia areata ; dermatitis herpetiformis : schleroderma ; vertiligo.

*c*) Those related to tuberculosis : erythema induratum ; lupus erythematosus ; lupus vulgaris ; lichen scrofulosorum.

*d*) Those having some relation to anaphylaxis eczema ; urticaria ; erythema multiforme ; angioneurotic edema.

*e*) Miscellaneous affections : rosacea ; granuloma annulare ; chilblains ; Raynaud's disease.

*f*) The presence of gingival infections are believed in many cases to supply the material for the infections of the tonsils and throat, these sites passing on the infection to those organs, tissues, and systems for which the tonsils have been held to be the infectious factor.

Q. 2. — **To what may be attributed the wide prevalence of gingival infections in the Army**?

A. —(1) The sum total of those factors which accompany the close association and aggregating of a large number of men with a diet and regimen different from that to which they have been accustomed.

(2) The lack of, or the inability or inopportunity to use, those toilet requisites necessary to exercise that care of the dental toilet to which men have been accustomed.

(3) The consumption of food, the greater amount of which is canned or preserved, the canning and preserving processes of which destroy in these foods their vitamines. The vitamines supply certain activating and fortifying elements to the body, the lack of which reduces the power of the physical organism to resist the invasion of micro-organisms, vast hosts of which are unavoidably found in and upon everything the men eat, drink, use or wear.

(4) The lack of these vitamines, together with the large ration of salt pork, bacon, ham, and salt beef, produces a nutritional disturbance which evidences itself in the mouth in a condition resembling a mild form of scurvy.

(5) Bacteriological examination of scurvy lesions in gums reveals micro-organisms similar to, if not the identical organisms of, Vincent's Angina.

(6) Another factor to be considered in this infective gingivitis is tobacco. More tobacco is used by men in military life than in civil life, the chronic irritation of which also lowers the resistance of the gum tissue and promotes the Vincent Angina micro-organisms.

(7) An insufficient amount of fresh vegetables and fresh fruit, the organic acids of which are prophylactic locally and systemically.

(8) Insufficient number of dental officers to remove the irritating dental factors that help to produce these gingival irritations.

(9) The lowered resistance of gingival tissues, which have previously been affected with pyorrhea alveolaris, renders easy and further promotes the irritating effects of micro-organisms, dietetic, hygienic, and dental factors.

(10) Therefore it is believed that the dietary conditions in, and the hygienic conditions of, military life prepare the soil for the invasion of the organisms of Vincent's Angina.

Q. 3. — **What methods may be adopted to combat these infections?**

A. — (1) The addition to the diet of the men of citrous fruit or such organic acids as will supply those organic acids which are prophylactic locally and systemically.

(2) A more liberal ration of tooth brushes and a dentifrice containing an organic acid.

(3) A more rigid and more frequent inspection of the mouth and teeth of the men to discover evidence of gingival irritation.

(4) A larger number of dental officers — one dentist to 500 men — to remove the local sources of gingival irritation.

(5) Well-organized drill for all soldiers on the toilet of the mouth.

(6) A more thorough sterilization of all cooking and eating ustensils and food supply.

Q. 4. — **What treatment gives the best results in eliminating the systemic effects of these infections?**

A. — When the gums are loose, hypertrophied, and where deep pus pockets are present, inject a local anesthetic, paint the gums with a strong solution of iodine, and excise with a small knife, or clip off with scissors the gum unattached to bone. Cut the gum clean down to the bone. Curet away the diseased tissue with small curet or spoon-shaped excavators between and around the teeth. Operate on one side of the mouth at a time. Where it is impossible to use instruments, destroy the pathological tissue with aromatic sulphuric acid, trichlora acetic acid, or sulphuric acid pure, neutralized in a minute with bicarbonate of soda. Paint all surfaces of all teeth and gums with a solution of iodine, iodide of potassium and iodide of zinc.

Remove as much as possible at this time of the deposits upon the teeth and polish with pumice stone to which a small amount of glycerine and sulphuric acid has been added. This is followed by an application of 10 o/o tincture of iodine. The patient is dismissed with instructions to use a dentifrice of 1 part vinegar, 1 part alcohol, and 8 parts water, as frequently as three or four times a day, or after eating and before going to bed.

The patient may be seen every other day and the gums painted with a 7 to 10 o/o tincture of iodine. When possible, spray the mouth with an antiseptic solution every day.

This treatment will so reduce the infection that within a short time the other side of the mouth may be treated surgically.

The establishment of proper elimination by use of calomel followed by a saline cathartic and a liberal amount of pure water, should be practiced; and for a week to ten days the diet should be restricted to vegetables and such foods as contain a low protein content. In those cases which may be characterized as superficial — in which the infection has not resulted in the formation of pockets between or about the teeth, the treatment may be begun by thoroughly removing all deposits, irritating fillings, etc., using a local anesthetic where necessary, and polishing with pumice to which sulphuric acid and glycerine have been added.

After this has been accomplished, paint the gums with a solution of 10 o/o tincture of iodine, to which has been added an equal amount of iodide of potassium and iodide of zinc crystals. It has also been advised to use an application of sulphuric acid, copper sulphate, peroxide of hydrogen, salvarsan, liq. potas. arsenitis, methylene blue or emetin. The use of chlorate of potash or potassium permanganate is recommended as a mouth wash.

1. The stimulation of the organs of elimination by the use of calomel, followed by saline cathartics and liberal amounts of pure water.

2. The addition to the diet of fresh vegetables and fresh fruit, such as lemons, oranges, limes, apples, etc.

3. Systemically — emetin.

4. The arsenical treatment.

Q. 5. — **Do focal infections about the apices devitalize tooth roots, and does cystic degeneration — the result of previous root infections — have any systemic or organic effects? What effect do they have on the jaws and sinuses?**

A. — A focus of infection is a circumscribed area of tissues infected with pathogenic micro-organisms, and when located in the jaws may produce effects upon different organs and systems of the body.

These effects may be produced in four ways : —

(1) By so lowering the systemic resistance that an invasion of another micro-organic host may establish a more serious infection.

(2) By the absorption of toxins.

(3) By the transfer of the micro-organism to some organ or system whose resistance has been reduced.

(4) By metastatic emboli of clumped micro-organisms which have become detached from some forces.

The most common effects of focal infections located in the jaws, together with focal infections located in other parts of the body are : — thyroid deficiency, infectious arthritis, leukemia, myositis, neuritis, goiter, asthma, nephritis, anemia, pernicious anemia, toxic hepatopathy, gastric and duodenal ulcer, appendicitis, chlolecystitis, endocarditis, myocarditis, pancarditis, hypertension, arterioschlerosis, chronic pharyngitis, pharyngeal abscesses, neuralgia, neuritis, acute mania, melancholia, dementia precox, hysteria major, neurasthenia, psychasthenia, epilepsy, lesions of the brain and spinal cord, bulbar paralysis.

Focal infections located in the jaws about the apices of diseased teeth have also been the cause of infections in the eye, ear, nose and necessary sinuses. Most of the effects that have been previously ascribed to gingival infections may also be caused by the apical infections in a more certain and insidious way, because the infection is pent up in the jaw bone, the absorption and distribution of which is constant.

### *Effects on the Jaws.*

(1) Gradual destruction of the bone, which may extend to such a degree as to destroy the vitality of one or several teeth.

(2) Production of cysts, which by extension may invade the nose or maxillary sinus.

(3) Cysts may so weaken the lower jaw as to produce pathological fracture.

(4) The chronic irritation of their pathological contents may result in malignancy.

(5) Infection of the glands of the neck.

Q. 6. — **What methods are employed to discover and eradicate these infections ?**

(1) As most of the infections occur on non-vital teeth, those in which there is any doubt about vitality may have their vitality tested after isolating with a rubber dam the tooth or teeth suspected.

*a*) By a spray of ethyl-chloride.

*b*) By a stream of ice water.

*c*) By a very hot instrument.

*d*) By a mild alternating electric current.

*e*) Transillumination.

*f*) Violet ray.

(2) As 75 o/o of non-vital teeth show evidence, in the radiograph, of infection, it is a reasonably safe proposition to suspect any dead tooth as a focus of infection.

(3) The radiograph may be used to discover if there is bone destruction about the root ends — evidence of infection.

(4) On the gums over suspected teeth, small scars may be looked for as evidence of previous fistulas leading to abscesses about the roots of the tooth or teeth suspected.

(5) All diseased and broken-down roots are to be suspected.

(6) The absence of teeth which cannot be accounted for, and which may be impacted, are to be searched for in the absence of radiographic evidence.

(7) The flap of gum which covers wholly or in part the lower third molar tooth may also be examined. This frequently is infected.

(8) Transillumination, in the absence of a radiograph, of the maxillary sinuses may reveal sources of infection connected with the upper bicuspid or molar roots.

(9) Enlargement of the submaxillary and sublingual glands may suggest some focus of tooth infection.

(10) Enlargement of the bone may be due to a hidden cyst, and this may be caused by some diseased tooth.

### *Eradication of Dental Focal Infections.*

(1) Eradication of the suspected tooth, curettement of the diseased tissue down to sound bone and healthy gum, providing for free drainage and self-cleansing, if possible, and protecting by packing with iodoform gauze until all surfaces are covered with health granulations.

(2) When the tooth is sufficiently valuable, and when two-thirds of the root is held by sound bone, the diseased root-end may be resected after sterilizing the root canal and properly filling it.

(3) In cases in which the gum flap over third molars is harboring infection, this flap must be cut away and the edges cauterized with sulphuric acid, phenol-sulphonic acid, trichloracetic acid painted with a tincture of iodine, and, where possible, packed with iodoform gauze for a few days to provide free drainage.

(4) In cases in which a cyst exists, the pathological tissue must be removed and the cavity packed with iodoform gauze until it has sufficiently filled in.

(5) Where the infection is due to an impacted tooth, this must be removed.

(6) In cases in which the maxillary sinuses have been invaded, the treatment depends upon the degree of infection. If it is slight, the removal of the infecting tooth and irrigation of the sinus with suitable non-irritating antiseptic, until the infection has subsided, are practised. When the infection has become chronic, it may be necessary to curet the sinus of all infected tissue, allowing it to fill in with new tissue.

Q. 7. — **Is the pulpless tooth a vital or non-vital organ?**

A. — The pulpless tooth is a non-vital organ, although it may be retained in its socket by the proper occlusion of the teeth, the shape of its root or roots, and by the attachment to the cementum of the peridental membrane.

Q. 8. — **Does a well-filled pulpless tooth cease to be a focus of infection?**

A. — The difficulty of sterilizing the tooth substance surrounding root canals and the area beyond the root-end, and keeping these surfaces sterile for any considerable time, on account of the tubular structure of the dentine, and the imperfection of the root canal fillings now used, renders the filled pulpless tooth a potential focus of infection, and the apical area a point of lowered resistance.

Q. 9. — **Will the loss of a painless infected tooth cause greater nutritional disturbance than the retention of the same tooth and focus of infection?**

A. — No. The retention of an infected tooth and focus of infection is a constant insidious source of infection, which is capable of producing effects so varied, and so remote in time and place from their origin, that when the problem is reduced to whether it is better to extract or not to extract, the answer is : — Extract and remove the focus of infection by direct surgical means.

